# Inadvertent Removal of a Right Ventricular Pacemaker Lead by a Knotted Transvenous Pacing Wire

**DOI:** 10.5005/jp-journals-10071-23126

**Published:** 2019-02

**Authors:** Evan J Wiens, Colette M Seifer, Clarence Khoo

**Affiliations:** 1 Department of Internal Medicine, Max Ready College of Medicine, University of Manitoba, Winnipeg MB, Canada; 2,3 Section of Cardiology, Department of Internal Medicine, University of Manitoba, Winnipeg, MB, Canada

**Keywords:** Knot, Loop, Right ventricular lead, Transvenous pacemaker

## Abstract

We report an unusual complication of permanent pacemaker (PPM) implantation in a patient with a temporary transvenous pacemaker (TVP) *in situ*, in which the TVP lead formed a knot around the newly-placed right ventricular (RV) lead, complicating TVP removal. The case underscores the fact that suboptimal TVP lead placement, such as looping in the RV, can result in complications during implantation of a permanent pacemaker.

**How to cite this article:**

Wiens EJ, Seifer CM *et al.* Inadvertent Removal of a Right Ventricular Pacemaker Lead by a Knotted Transvenous Pacing Wire. Indian J of Crit Care Med 2019;23(2):102-103.

## INTRODUCTION

Temporary transvenous pacing is commonly used in the acute management of bradyarrhythmias as a means of maintaining hemodynamic stability while awaiting insertion of a permanent pacemaker (PPM)^1^. Reports exist of intracardiac devices forming knots, and this can significantly complicate their removal^2^. This is most commonly seen with Swan-Ganz catheters^3^. However, such reports with TVP wires are distinctly uncommon, with only one recent report describing a knotted TVP which significantly complicated removal of the device.^4^ However, no reports exist to our knowledge of TVP wire forming a knot with a separate intracardiac device. Here, we report an unusual complication of PPM implantation in a patient with a TVP *in situ*, in which the TVP lead formed a knot around the newly-placed right ventricular (RV) lead, complicating TVP removal.

### Case Report

**Fig. 1:**
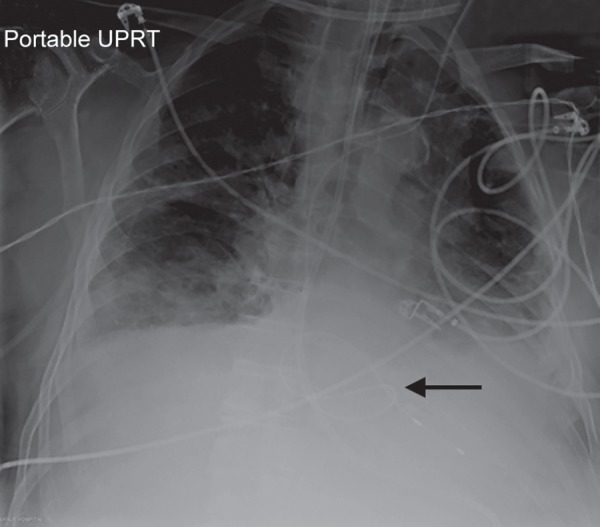
Following TVP insertion, the looped lead is visible in the RV on chest X-ray (arrow)

A 79-year-old male with a history of chronic obstructive pulmonary disease, type 2 diabetes, chronic kidney disease, and persistent atrial fibrillation was brought to hospital after being successfully resuscitated following a brief pulseless electrical activity (PEA) cardiac arrest. During his convalescence, he had a second PEA arrest, from which he was again successfully resuscitated. Telemetry revealed atrial flutter with atrioventricular conduction that slowed markedly to ventricular rates as low as 34 beats per minute. A balloon-tipped temporary pacing catheter was floated in via left internal jugular vein until adequate ventricular capture was observed. Fluoroscopy was not used during insertion. A follow-up chest X-ray confirmed appropriate placement of the lead, which revealed the lead in the right ventricle with redundant lead slack forming a loop (Fig. 1).

The patient subsequently had a PPM implanted. The PPM was implanted without complication using a standard left cephalic vein access. The lead was advanced under fluoroscopic guidance. Implantation of the PPM lead was carried out without difficulty with active fixation to the right ventricular septal wall. The lead pin was attached to a pacemaker pulse generator which was then placed in a pocket located in the prepectoral plane below the left subclavicular fossa.

**Figs. 2A and B:**
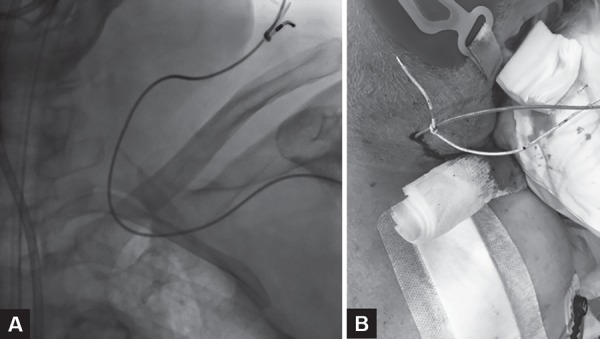
The snared RV lead is visible fluoroscopically before (A) and photographically after (B) inadvertent removal through the skin overlying the left internal jugular vein

The TVP was then removed under fluoroscopic guidance. The large loop of redundant wire was noted, but it exited the cardiac silhouette without any resistance or interaction with the newly implanted PPM lead. However, significant resistance was noted when attempting to pull the tip of the TVP lead out through the introducer sheath. This resistance was only encountered when the TVP lead tip was at the level of the skin; fluoroscopy was not performed at this stage to determine the cause of resistance. The resistance was overcome and the TVP lead was successfully removed. It quickly became evident that the reason for the resistance was that the TVP lead had looped and formed a knot around the RV PPM lead at the junction of the left subclavian and left internal jugular vein. Continued traction had thus dislodged the RV lead tip from the endocardium, resulting in inadvertent removal of the RV lead through the left internal jugular vein (Fig. 2). No dislodgement of the PPM lead had been apparent fluorocopically during removal of the TVP wire from the cardiac silhouette as the ensnarement occurred at the level of the thoracic inlet when fluoroscopy was no longer being employed, and no loss of capture was noted as the patient was in their intrinsic rhythm. The patient remained hemodynamically stable.

In order to place a new RV PPM lead, left axillary venous access was obtained and a new lead was successfully placed, which was connected to the original pulse generator. The ensnared RV lead was cut within the pocket, and the lead pin was removed from the pulse generator directly. The external portion of the RV lead (Fig. 2B) was then removed without complication from the left internal jugular vein. The new PPM assembly functioned well, and the procedure was completed without any further complication.

## DISCUSSION

This case represents a previously unreported complication of PPM implantation in patients with a TVP *in situ*. Although this patient did not suffer any deleterious effects, it is possible that vascular or valvular injury, infection, symptomatic loss of pacing or hemodynamic compromise might result from events such as this. In addition, if a permanent lead utilizing active fixation is deployed either at the apex or the ventricular free wall, it is possible that inadvertent removal of the lead while the fixation helix is still deployed could result in traumatic avulsion of a portion of the attached myocardium and the subsequent development of a hemopericardium.

This case illustrates that care must be taken when removing a TVP lead following PPM implantation, and underscores the fact that suboptimal TVP lead placement, such as looping in the RV, can result in complications. Such loops may be avoided by placement of TVP under fluoroscopy if feasible. If such loops are discovered subsequently, as in this case, attempts to reduce the loop may prevent such complication.

If a loop in a TVP is identified during PPM implantation, then reduction of the loop should be performed prior to introduction of a permanent pacing lead. Alternatively, the TVP loop should be closely observed during TVP removal to ensure that a knot does not form around the pacing lead. In this case, despite careful fluoroscopic observation during TVP removal, snaring and removal of the PPM lead still occurred. This illustrates the importance of prevention and reduction of such loops, if possible.

## CONCLUSION

Permanent pacemakers are often placed in patients with temporary transvenous pacemakers *in situ.* Suboptimal TVP lead placement, such as looping in the RV, can complicate PPM insertion and result in potential adverse events such as valvular or vascular injury, symptomatic loss of pacing or hemodynamic compromise. Such loops should be avoided or reduced if possible, or closely observed fluoroscopically during TVP removal to ensure that a knot does not form around the pacing lead.
